# Ventricular septal rupture after blunt chest trauma: a case report

**DOI:** 10.1186/s40792-022-01448-z

**Published:** 2022-05-12

**Authors:** Masato Tochii, Hirotaka Watanuki, Kayo Sugiyama, Yasuhiro Futamura, Hiroshi Ishikawa, Katsuhiko Matsuyama

**Affiliations:** 1 Department of Cardiac Surgery, Aichi Medical University, 1-1 Yazakokarimata, Nagakute, Aichi 480-1195 Japan; 2 Department of Cardiac Surgery, Kasugai Municipal Hospital, 1-1 Takagi, Kasugai, Aichi 486-8510 Japan

**Keywords:** Ventricular septal rupture, Trauma

## Abstract

Cardiac injury, including myocardial contusion and valvular damage, is a common complication of blunt chest trauma; however, traumatic ventricular septal rupture is a rare complication. We encountered a rare case of ventricular septal rupture following blunt chest trauma that was successfully repaired by emergency surgery. The mechanism underlying rupture may involve acute compression of the heart between the sternum and the vertebral column when the ventricle is filled, thereby causing a sudden increase in intraventricular pressure and leading to septal rupture. Emergency operation should be considered in cases of large defects and hemodynamic instability.

## Background

The incidence of ventricular septal rupture (VSR) after blunt chest trauma (BCT) remains unknown in the literature[1–3]. VSR is reported to occur because of compression of the heart between the sternum and the vertebral column, causing a sudden increase in intraventricular pressure at the time of atrioventricular valve closure, due to BCT [4]. However, BCT rarely causes VSR as a complication. The clinical presentation may be acute, subacute, or late depending on the extent of the injury and subsequent local necrosis [2, 3]. Surgical or interventional closure is necessary in patients with hemodynamic instability or large defects. We present a rare case of a large VSR after BCT, without any other myocardial damage, that was successfully closed with emergent surgery.

## Case presentation

**Fig. 1:**
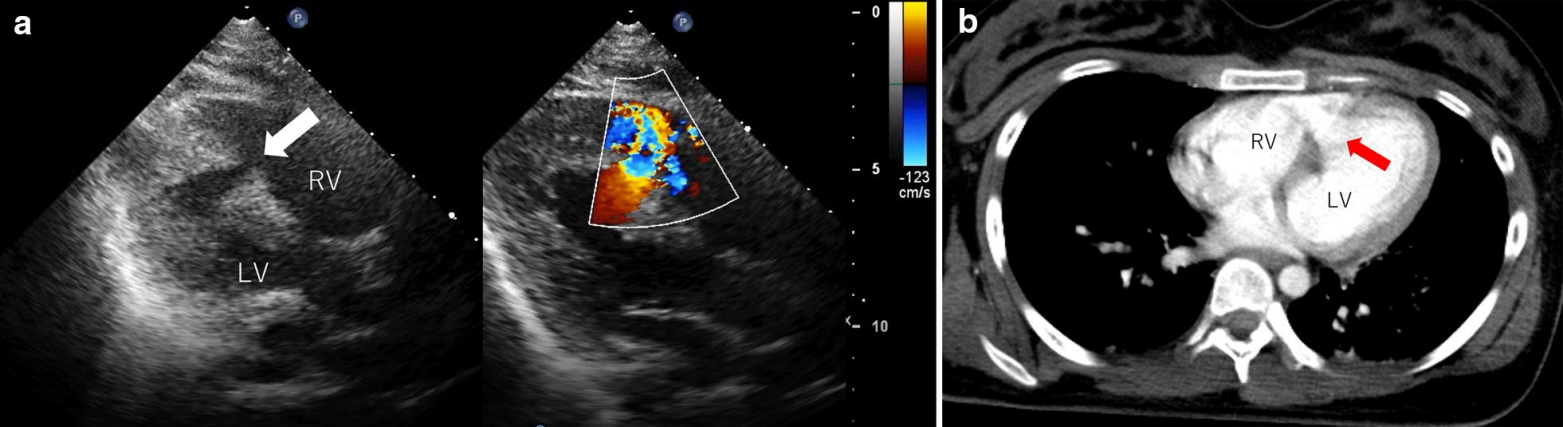
**a** Echocardiography findings showing ventricular septal rupture (arrow) at the muscular septum, and a left-to-right shunt with a pulmonary-to-systemic flow ratio of 4:1. **b** Chest computed tomography findings indicating defects in the ventricular septum (arrow). LV: left ventricle, RV: right ventricle

**Fig. 2:**
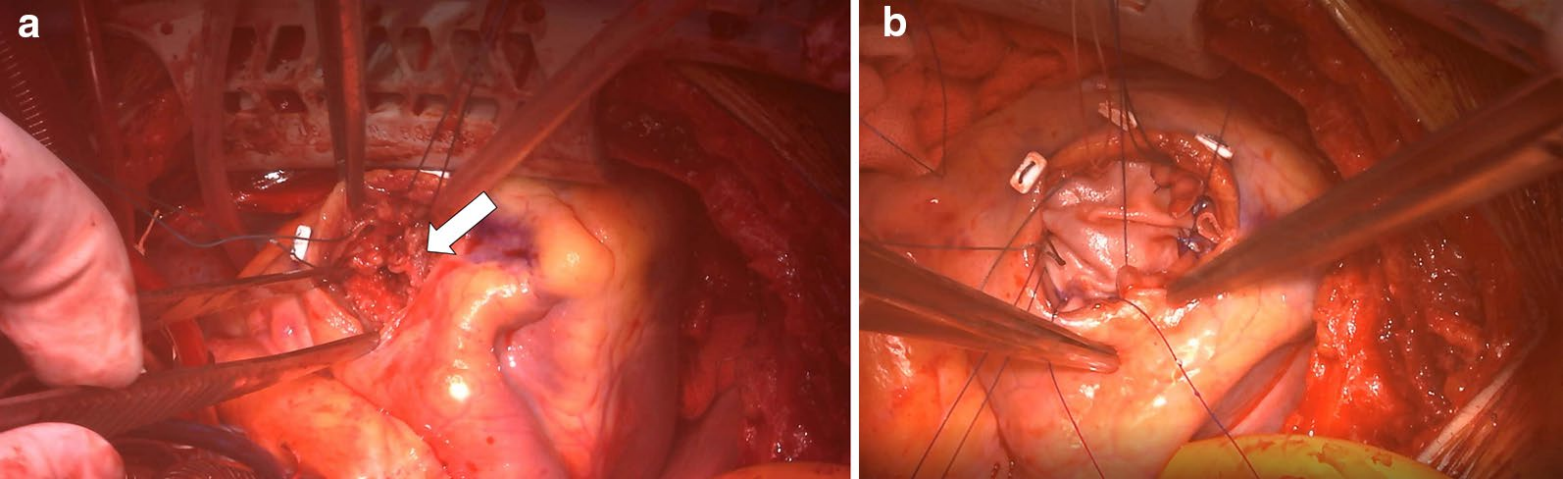
**a** Inspection of the defect through an incision in the right ventricle, located at the muscular ventricular septum (arrow). **b** Closure of the defect with two pieces of bovine pericardial patches, first patch in the left ventricle and second patch in the right ventricle

A healthy 18-year-old female patient was admitted to our hospital after a traffic accident. She stepped on the seat next to the driver, and a large automobile accident occurred as a car overturned. Despite the large accident, a whole-body computed tomography scan demonstrated no obvious damage to her head and abdominal organs. She had no fractures and was able to walk; the hemodynamics were also stable. Approximately 6 h after the accident, she developed unstable hemodynamics, low systemic oxygen saturation, and progressive pulmonary edema. Endotracheal intubation was performed, and mechanical ventilation was initiated. Although echocardiography at the timing of admission detected no obvious abnormal findings, transthoracic echocardiography by the cardiologist after the endotracheal intubation showed a 1 × 2 cm large rupture of the midmuscular ventricular septum and a left-to-right shunt with a pulmonary-to-systemic flow ratio of 4:1. And the diagnosis of VSR on computed tomography confirmed retrospectively after the diagnosis on echocardiography (Fig. 1). An emergency operation was planned, and high-dose inotropic agents and intra-aortic balloon pumping were administered for the unstable hemodynamics. The right ventricle was greatly expanded, but no myocardial contusions were observed when the chest was opened through a median sternotomy. Cardiopulmonary bypass was established, with aortic and bicaval cannulation. A vent tube was placed through the right superior pulmonary vein to prevent cardiac distension. The ascending aorta was cross-clamped, and myocardial protection was performed with perfusion using cold blood cardioplegia. A 5-cm-long right ventricular incision was made 2 cm away from and parallel to the left anterior descending artery. The VSR, with unhealthy margins, was found to involve the muscular septum (Fig. 2). The defect (2 × 2 cm) was closed with two pieces of bovine pericardial patches (5 × 5 cm) using 10 interrupted, pledgetted horizontal mattress 3–0 polypropylene sutures placed on the healthy ventricular septal wall, exercising caution to avoid damage to the papillary muscle. The polypropylene suture was first inserted into the first pericardial patch, then through the ventricular septum from left to right, and finally through the second patch. The first patch was placed in the left ventricle, and the second patch was placed in the right ventricle. The right ventricular incision was closed using 3–0 polypropylene sutures with Teflon-felt strip reinforcement. The patient could not be weaned off cardiopulmonary bypass because of lung edema and poor hemodynamics, especially due to right ventricular damage because of the greatly expanded myocardium preoperatively. Cardiopulmonary bypass was converted to extracorporeal membranous oxygenation using central cannulation of the ascending aorta and bicaval drainage. She was transferred to the intensive care unit, with a chest opening and mechanical cardiopulmonary support. Until the 3 days after the initial operation, the heart was stand still like stone heart. She recovered over the next seven days and was successfully weaned from mechanical cardiopulmonary support and intra-aortic balloon pumping. The chest was closed and extubated 8 days after the first operation, without any neurological deficits. Echocardiography showed good biventricular function and no residual shunt across the ventricular septum.

## Discussion

Cardiac injury is a common complication of BCT [2–5]. The common injuries include cardiac contusion, valvular damage, and aortic insufficiency [2, 3]. Myocardial contusion is the most frequent cardiac injury following BCT and occurs in 16–76% of patients in motor vehicle accidents; however, traumatic VSR is a rare complication of BCT [1, 4, 6]. VSR may be delayed for several hours to several days after trauma, and its clinical presentation varies from immediate death to complete spontaneous closure [7]. The most common localization of traumatic VSR is in the muscular portion of the interventricular septum near the cardiac apex [3, 5, 7]. In the present case, the patient had no history of congenital heart disease.

The first postulated mechanism of VSR is acute compression of the heart between the sternum and the vertebral column, with a resultant sudden rise in intracardiac pressure at the end of diastole or isovolumetric systole. The second postulated mechanism is microvascular disruption caused by myocardial injury, leading to infarction and liquefaction of the septum [1, 2, 4]. The contused myocardium can become necrotic and, subsequently, perforate. Therefore, VSR may occur several hours or months after BCT [7]. The former postulation was considered to be the underlying mechanism in this case, because the rupture occurred in the acute phase and there was no myocardial infarction around the defect. The high pressure of compressed and filled left ventricle caused the VSR in this case; however it is the pressure toward the free wall of left ventricle that caused the fatal cardiac rupture. Right ventricle is more vulnerable site rather than left ventricle. However, in this situation, myocardial damage should have occurred in the left side of the heart not to right side because the pressure of right ventricle is quite low. A conservative approach has been recommended for patients with stable hemodynamics and small defects when the ratio of pulmonary-to-systemic flow is less than 2:1 because spontaneous closure may occur [3, 7]. Echocardiography is a useful method for the diagnosis of VSR, however in the presented case, examination of the heart at the admission detected no VSR. If it is a large automobile accident, computed tomography and echocardiography should be examined in detail, keeping in mind the possibility of cardiac injury even if the hemodynamics are stable for the first time. The only way to treat a large defect and a patient with unstable hemodynamics is prompt diagnosis and emergent operation.

We approached the VSR through the right ventricle, not the left ventricle, to prevent left ventricular function. In the case of VSR after myocardial infarction, we used a large patch to close the infarcted myocardium, the so-called infarct exclusion technique [8], which is placed from inside of the ventricular cavity to the free wall with viable myocardium just to the coronary artery to avoid cutting and residual shunting. In this case, a double patch was used to close the defect. Interrupted, pledgetted horizontal mattress sutures were placed on the healthy ventricular septal wall and not on the left ventricular anterior wall [9]. We think the right ventricular approach and the double-patch method is a suitable and standard technique for this situation.

## Conclusions

In summary, we present a rare case of traumatic VSR following a car accident that was successfully repaired by an emergency operation, despite a troublesome postoperative course. Traumatic VSR occurs in both the acute and chronic phases, and prompt diagnosis is mandatory for this often-fatal complication.

## Data Availability

Not applicable.

## Abbreviations

VSR: Ventricular septal rupture
BCT: Blunt chest trauma
